# Roles of Autophagy, Mitophagy, and Mitochondria in Left Ventricular Remodeling after Myocardial Infarction

**DOI:** 10.31083/RCM28195

**Published:** 2025-03-24

**Authors:** Xin Zhang, Shuai Shao, Qiuting Li, Yi Wang, Mowei Kong, Chunxiang Zhang

**Affiliations:** ^1^Department of Cardiology, The Affiliated Hospital of Southwest Medical University, 646000 Luzhou, Sichuan, China

**Keywords:** left ventricular dysfunction, myocardial infarction, autophagy, ventricular remodeling, heart failure, therapeutic strategies

## Abstract

This review examines the mechanisms of left ventricular dysfunction, focusing on the interplay between ventricular remodeling, autophagy, and mitochondrial dysfunction following myocardial infarction. Left ventricular dysfunction directly affects the heart’s pumping efficiency and can lead to severe clinical outcomes, including heart failure. After myocardial infarction, the left ventricle may suffer from weakened contractility, diastolic dysfunction, and cardiac remodeling, progressing to heart failure. Thus, this article discusses the pathophysiological processes involved in ventricular remodeling, including the injury and repair of infarcted and non-infarcted myocardia, adaptive changes, and specific changes in left ventricular systolic and diastolic functions. Furthermore, the role of autophagy in maintaining cellular energy homeostasis, clearing dysfunctional mitochondria, and the key role of mitochondrial dysfunction in heart failure is addressed. Finally, this article discusses therapeutic strategies targeting mitochondrial dysfunction and enhancing mitophagy, providing clinicians and researchers with the latest insights and future research directions.

## 1. Introduction

The left ventricle, as the primary pumping chamber of the heart, is responsible for delivering oxygen and nutrients throughout the body [1]. Its contractile and diastolic functions directly impact the heart’s pumping efficiency and are key indicators for assessing cardiac performance [2]. The normal function of the left ventricle is crucial for the health of the cardiovascular system and its dysfunction can lead to severe clinical consequences, including arrhythmias, heart failure, and sudden death. Therefore, maintaining and improving left ventricular function is essential for the management of cardiovascular diseases [3, 4].

Myocardial infarction (MI) is a severe disease caused by the acute occlusion of the coronary arteries, leading to ischemic necrosis of myocardial cells [5]. It causes local myocardial tissue damage and also triggers complex biological reactions that affect the infarcted area, the entire left ventricle [6, 7]. Consequently, left ventricular function may be severely compromised, characterized by weakened contractility, diastolic dysfunction, and cardiac remodeling [8]. These alterations can eventually progress to heart failure, severely affecting patients’ quality of life and prognosis [9]. The death of myocardial cells after myocardial infarction triggers a series of complex biological reactions, including inflammation, apoptosis, and ventricular remodeling, which collectively affect left ventricular function [10].

Autophagy, particularly mitophagy, is integral to maintaining cellular energy homeostasis and facilitating the removal of dysfunctional mitochondria [11]. Autophagy is a lysosome-mediated pathway that helps eliminate damaged organelles, and mitophagy specifically targets mitochondria, which is essential for optimizing cardiac function [12]. Enhancing autophagy and mitophagy can combat ischemic heart disease and improve cardiac function after myocardial infarction [13, 14]. For example, berberine promotes autophagy and reduces left ventricular remodeling and dysfunction after myocardial infarction [15]. Under hypoxic conditions, FUN14 domain-containing protein 1 (Fundc1)-mediated mitophagy helps restore mitochondrial function and protect cardiomyocytes from ischemic injury [16]. Therapeutically improving mitophagy may be an effective strategy for treating heart failure and improving outcomes after myocardial infarction. For instance, studies have shown that treatments inhibiting mitochondrial oxidative stress or promoting Fundc1-mediated mitophagy show promise in preventing heart failure and improving cardiac function [17, 18].

This review explores the mechanisms underlying left ventricular dysfunction following myocardial infarction, with a particular focus on ventricular remodeling, autophagy, and mitochondrial dysfunction. By integrating current scientific literature and recent research advancements, it offers clinicians and researchers the latest insights into the pathophysiology of left ventricular dysfunction, therapeutic strategies, and directions for future research.

## 2. Changes in Left Ventricular Function after Myocardial Infarction

### 2.1 Pathophysiological Process of Ventricular Remodeling

#### 2.1.1 Injury and Repair of Infarcted Myocardium

**Fig. 1.**
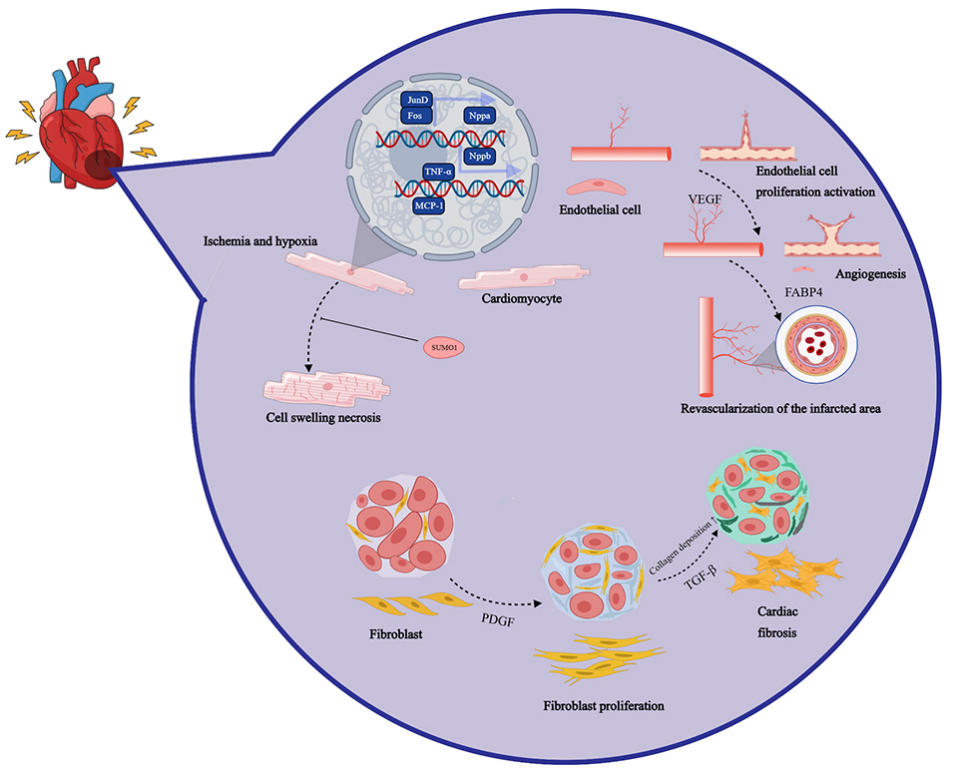
**After myocardial infarction, the cardiac injury repair process is a complex multi-stage pathophysiological process involving inflammation, neovascularization, and myocardial regeneration**. Nppa, natriuretic peptide A; SUMO1, small ubiquitin-like modifier 1; VEGF, vascular endothelial growth factor; FABP4, fatty acid binding protein 4; TGF-β, transforming growth factor beta; MCP-1, monocyte chemoattractant protein-1; Jund, JunD proto-oncogene; Nppb, natriuretic peptide B; Fos, Fos proto-oncogene; CUMOL, cumene; TGF, transforming growth factor; PDGF, platelet-derived growth factor.

After myocardial infarction, the cardiac injury repair process is a complex multi-stage pathophysiological process involving inflammation, neovascularization, and myocardial regeneration [19]. In the acute phases following myocardial infarction, neutrophils rapidly infiltrate the infarct area, triggering local inflammation and releasing matrix metalloproteinases (MMPs), neutrophil elastase (NE), and other enzymes that help phagocytize and remove dead myocardial cells and matrix debris [20, 21]. Subsequently, monocytes and M1-type macrophages are recruited to the infarct area, expressing pro-inflammatory factors such as tumor necrosis factor (TNF), further intensifying the inflammatory response [22]. In the late stage of inflammation, neutrophils undergo apoptosis, promoting macrophage polarization and transformation into anti-inflammatory M2-type macrophages, which express anti-inflammatory, profibrotic, and vascular endothelial growth factor (VEGF), promoting the resolution of inflammation [23, 24]. These cytokines and growth factors collectively stimulate the proliferation of fibroblasts and endothelial cells, initiating the cardiac repair phase. This process involves the formation of new blood vessels and a temporary extracellular matrix (ECM), ultimately leading to scar tissue formation, promoting myocardial fibrosis, and mitigating myocardial rupture [25, 26]. Left ventricular remodeling and dysfunction are the primary complications following myocardial infarction, characterized by left ventricular cavity dilation, wall thinning, and contractile dysfunction [27, 28]. For example, compared with sham-operated rats, the left ventricular ejection fraction (LVEF) of rats after myocardial infarction decreased by an average of 61%. Overexpression of peroxiredoxin-3 (Prx-3) has been shown to inhibit left ventricular remodeling and heart failure by reducing myocardial cell hypertrophy, interstitial fibrosis, and apoptosis [29]. The inflammatory response triggered by myocardial infarction aims to clear necrotic debris, followed by an anti-inflammatory repair phase [30]. An imbalance in inflammation regulation may exacerbate the infarct area, promote myocardial cell death and impair contractile function [31]. Elevated levels of C-reactive protein are associated with poor prognosis in patients with acute myocardial infarction (AMI) and heart failure (HF) [25, 28]. The process of injury and repair of the myocardium in the infarct area is shown in Fig. 1.

Reperfusion of surviving myocardium can improve cardiac function and alleviate symptoms of heart failure. Pharmacological treatment targeting the renin-angiotensin-aldosterone system and β-adrenergic receptors can reduce myocardial fibrosis, hypertrophy, and adverse left ventricular remodeling [32]. Anti-inflammatory treatments, such as interleukin-1 β (IL-1β) monoclonal antibody and low-dose colchicine, have shown efficacy in reducing adverse cardiovascular events after myocardial infarction [33]. Mitochondrial dysfunction is a key factor in ischemic injury and heart failure [34]. After myocardial infarction, a reduction in mitochondrial DNA copy number and enzyme leads to cell death and impaired cardiac function. Interventions targeting mitochondrial oxidative stress, such as Prx-3 overexpression, can ameliorate these effects [35, 36]. Cardiac remodeling and heart failure are the main drivers of chronic heart failure after myocardial infarction, caused by sustained ischemic injury, leading to myocardial damage, oxidative stress, inflammation, and mitochondrial dysfunction [37, 38]. These factors collectively promote adverse left ventricular remodeling, increase wall stress, and impair cardiac function.

#### 2.1.2 Adaptive Changes in Non-Infarcted Myocardium 

Adaptive changes in non-infarcted myocardium following myocardial infarction significantly impact cardiac function and remodeling processes. These changes include myocardial cell hypertrophy, apoptosis, and diffuse fibrosis, which collectively act to maintain the overall function of the heart. Initially, myocardial cell hypertrophy is a key feature of ventricular remodeling, involving an increase in the volume of non-infarcted myocardial cells to compensate for the lost contractility in the infarcted area [39, 40]. This hypertrophy, while initially helpful in maintaining cardiac pumping function, ultimately leads to myocardial cell dysfunction and further deterioration of cardiac function [41, 42]. Enlarged myocardial cells experience disruptions in energy metabolism, leading to a decline in contractile function. This process resembles the gene regulation observed in fetal and early neonatal cells, which grow in a more elongated shape. However, these embryonic isoforms are prone to fatigue, accelerating myocardial cell dysfunction and reducing their life span [43]. Secondly, fibrosis in non-infarcted myocardium is also an important feature of ventricular remodeling. The increased fibrotic tissue leads to increased heart stiffness, affecting the heart’s diastolic and systolic functions [44]. After myocardial infarction, collagenase activation in the infarct area, collagen fiber degradation, and insufficient collagen matrix contributes to progressive thinning of the infarct area, and ventricular dilation [45]. Conversely, increased collagen content can stiffen the ventricular wall, reduce ventricular compliance, and result in diastolic dysfunction [46].

Additionally, autophagy in non-infarcted myocardium plays a crucial role in ventricular remodeling. By removing damaged organelles and proteins, autophagy helps maintain cellular homeostasis and is closely linked to processes such as growth, development, differentiation, and aging. However, its dysregulation can contribute to myocardial cell death and various diseases [47, 48]. In ventricular remodeling, left ventricular dilation, increased wall stress, and changes in cardiac geometry are also important pathophysiological changes [49]. These changes cause the heart to shift from an elliptical to a spherical shape leading to increased myocardial wall mass and left ventricular enlargement. Additionally, dysfunction in non-infarcted myocardium may arise due to variations in local wall stress distribution, which can trigger fundamental biochemical and cellular alterations, such as myocardial hypertrophy and apoptosis [50].

### 2.2 Specific Changes in Left Ventricular Function

#### 2.2.1 Changes in Systolic Function

Myocardial infarction significantly affects the systolic function of the left ventricle, leading to a decrease in LVEF. This decrease directly reflects a weakening of myocardial contractility. LVEF is an important indicator for assessing left ventricular systolic function, and its decrease means reduced cardiac pumping capacity and cardiac output [51]. Myocardial cell necrosis and stunning contribute to left ventricular systolic dysfunction, and local wall motion abnormalities are also prevalent, which may suggest potential coronary heart disease, Takotsubo syndrome, or myocarditis [52]. Echocardiography is the preferred method for assessing cardiac structure and systolic function, providing information about left ventricular hypertrophy and local wall motion abnormalities [53]. Study has shown that guided exercise rehabilitation can significantly improve left ventricular systolic function in patients with AMI, with a significant decrease in N-terminal pro-brain natriuretic peptide (NT-proBNP) levels in the exercise group and a stable left ventricular diastolic diameter (LVDd). In contrast, the control group showed an increase in LVDd [54]. Moreover, early cardiac rehabilitation and timely revascularization, including percutaneous coronary intervention (PCI), can reduce myocardial scar and improve LVEF by preventing myocardial cell apoptosis and promoting the repair of ischemic myocardium [55]. Stent implantation significantly reduces left ventricular volume in AMI patients and improves systolic and diastolic functions over time [56]. PCI performed within 12 hours of the onset of AMI can significantly reduce brain natriuretic peptide (BNP) levels and improve left ventricular function. When compared to conservative treatment, selective PCI demonstrates greater efficacy in enhancing LVEF while reducing left ventricular end-systolic volume (LVESV) and end-diastolic volume (LVEDV) [57]. Additionally, a history of angina, particularly pre-infarction angina, is associated with better collateral circulation in the coronary arteries and higher LVEF after AMI, thereby offering protective effects for cardiac function [58]. Myocardial remodeling after AMI involves an increase in myocardial cell proliferation and actin expression, helping hypertrophy and functional maintenance in non-infarcted areas [59]. Quantitative tissue velocity imaging (QTVI) effectively assesses left ventricular systolic dysfunction and in AMI patients, with significant differences in end-diastolic volume (EDV), end-systolic volume (ESV), and ejection fraction (EF) compared to healthy individuals [60]. Thrombolytic therapy helps reperfusion of the infarct-related artery, reduces infarct size, limits left ventricular remodeling, and maintains cardiac function [61]. Basic mitral regurgitation (MR) and its development after AMI are related to left ventricular size, shape, and function, affecting cardiovascular outcomes [62]. An increase in VEGF and granulocyte colony-stimulating factor (G-CSF) levels after PCI is related to the improvement of LVEF, indicating their role in myocardial repair and neovascularization [63]. AMI patients with ventricular arrhythmias show reduced heart rate variability and reduced LVEF, with an increased incidence of early repolarization and fragmented QRS waves [64]. Autologous bone marrow stem cell transplantation enhances segmental myocardial function and LVEF following AMI, as assessed by tissue tracking and myocardial perfusion imaging [65]. Additionally, rescue PCI after failed thrombolysis significantly improves cardiac function compared to delayed PCI, with benefits sustained for at least six months [66].

#### 2.2.2 Impact on Diastolic Function

Diastolic dysfunction after myocardial infarction is a complex process involving changes in cardiac structure and function. This injury is often manifested as increased left ventricular filling pressure, affecting cardiac filling and diastolic end-pressure [67]. Diastolic dysfunction is closely related to the severity and prognosis of heart failure and is an indispensable part of heart failure treatment. Cardiac magnetic resonance (CMR) imaging and echocardiography play important roles in assessing left ventricular diastolic function [68]. Strain analysis of CMR can detect early systolic and diastolic strain injuries, providing a new perspective on the assessment of diastolic function [69]. Echocardiographic indicators for assessing left ventricular diastolic function are divided into primary and secondary indicators, including mitral valve diastolic flow velocity (E peak, A peak) and mitral valve E peak deceleration time (DT) [70]. These indicators help determine filling types and further judge filling pressure related to prognosis.

Echocardiography, as a non-invasive and simple means, can effectively reflect cardiac function. However, there are certain defects in the commonly used ultrasound indicators during ventricular remodeling after myocardial infarction. Cardiac remodeling is a global phenomenon that also involves the left atrium and the mitral apparatus. Different remodeling patterns have been identified, showing that cases with increased left ventricular end-diastolic volume index (LVEDVI) and normal left ventricular end-systolic volume index (LVESVI) have the smallest infarct size and better hemodynamics compared to cases with increased LVESVI and normal LVEDVI. The current patterns of cardiac remodeling are not clearly defined after myocardial infarction, which limits the application of echocardiographic imaging in assessing the severity of remodeling [71]. The Tei index, as a new evaluation index, can comprehensively evaluate the systolic and diastolic functions of the ventricles and is not affected by cardiac geometry, heart rate, blood pressure, *etc* [72].

#### 2.2.3 Cardiac Remodeling and Functional Failure

**Fig. 2.**
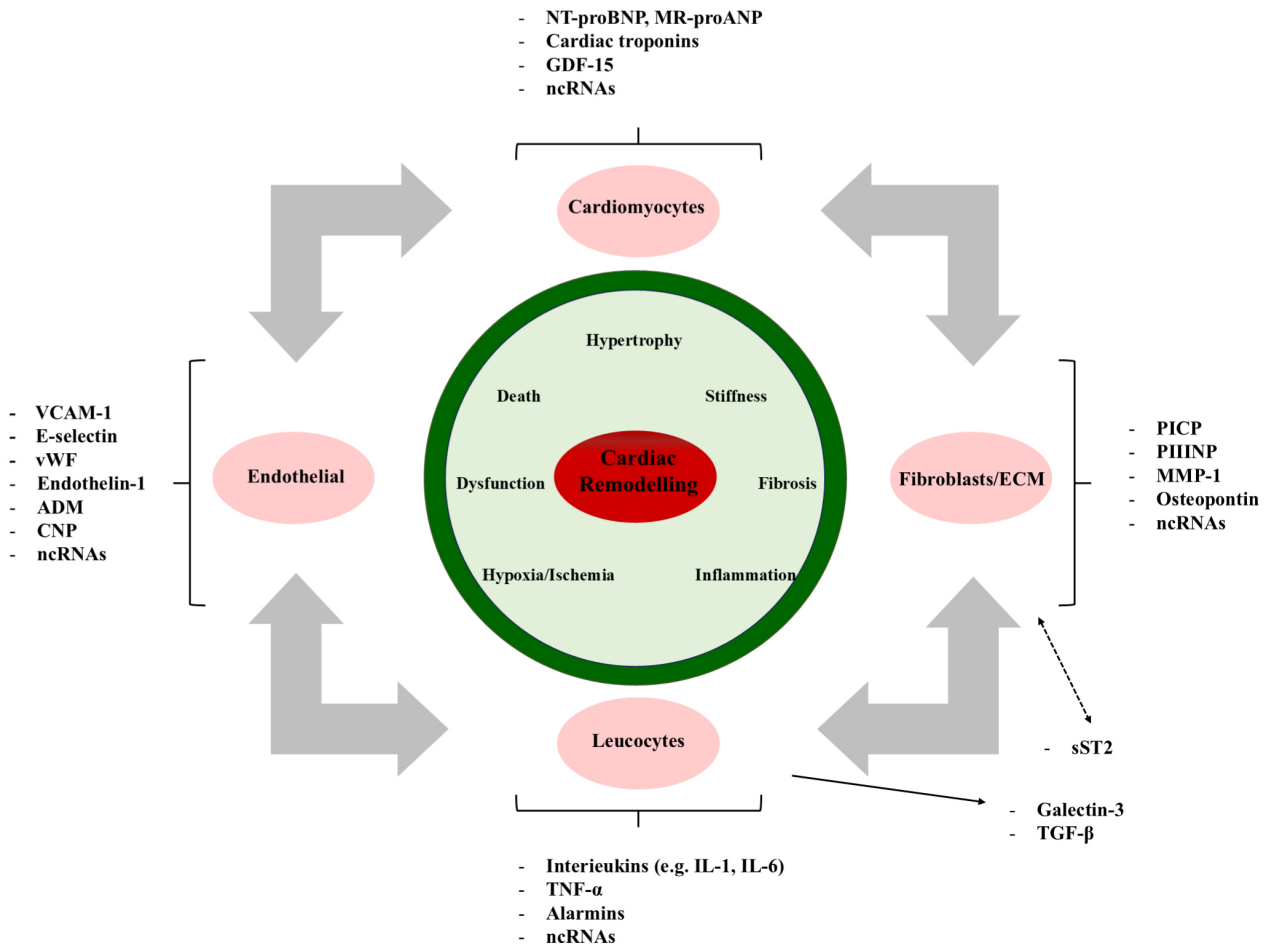
**The molecular mechanisms of cardiac remodeling involve various pathophysiological stimuli**. VCAM-1, vascular cell adhesion molecule-1; ADM, adrenomedullin; CNP, C-type natriuretic peptide; BNP, B-type natriuretic peptide; GDF-15, growth differentiation factor 15; EVs, extracellular vesicles; PIIINP, procollagen III N-terminal propeptide; MMP-1, matrix metalloproteinase-1; TNF-α, tumor necrosis factor alpha; PICP, procollagen I C-terminal propeptide; CITP, chronic immune thrombocytopenia; sST2, soluble suppression of tumorigenicity 2; IL, interleukin; ncRNA, non-coding RNA; vW, von willebrand; NT-proBNP, N-terminal pro-brain natriuretic peptide; MR-proANP, midregional pro-attrial natriuretic peptide; TGF-β, transforming growth factor-β; ECM, extraCellular matrix.

Cardiac remodeling after myocardial infarction is a complex process involving changes in the size, shape, structure, and function of the heart, including left ventricular enlargement, reduced LVEF, and local wall motion abnormalities [73, 74]. Cardiac remodeling not only affects the redistribution of myocardial cells, interstitial cells, and blood vessels but also leads to increased heart stiffness and decreased contractility [75, 76]. The latest research indicates that in the process of cardiac remodeling, myocardial cell hypertrophy, apoptosis, fibrosis remodeling, vascular remodeling, and electrical signal remodeling all play a role [77, 78]. Cardiac remodeling is the main factor determining the incidence and long-term prognosis of cardiac events after myocardial infarction and is an independent predictor of heart failure [79, 80]. Loss and dysfunction of cardiomyocytes due to oxygen deprivation or lack of blood flow can initiate a series of events leading to cardiac remodeling. This process is marked by the pathological enlargement of cardiomyocytes, regulated by pathways like calcium-dependent excitation-transcription coupling [78]. Additionally, cardiac fibrosis, characterized by the abnormal accumulation of extracellular matrix, is a key part of remodeling, mainly driven by cardiac fibroblasts [72, 78]. Inflammation also plays a significant role, with white blood cells contributing to remodeling through the release of cytokines and chemokines [78]. The molecular mechanisms of cardiac remodeling are shown in Fig. 2.

Prevention and treatment strategies for cardiac remodeling include pharmacological treatment, interventional treatment, and lifestyle adjustments, aiming to improve cardiac structure and function, prevent or reverse cardiac remodeling, and reduce the occurrence and development of heart failure [79]. Recent research indicates that the novel aminopeptidase A inhibitor Firibastat is not superior to ramipril in preventing left ventricular dysfunction after acute myocardial infarction. However, it has a comparable safety profile, effectively inhibits angiotensin II, and may contribute to improved myocardial remodeling [80]. In addition, the molecular mechanisms of cardiac remodeling involve various pathophysiological stimuli, including myocardial cell loss, cardiac hypertrophy, changes in extracellular matrix homeostasis, fibrosis, autophagy deficiency, metabolic abnormalities, and mitochondrial dysfunction [81]. These processes may initially serve as protective compensatory mechanisms for the heart, but if prolonged, they can lead to the progression of heart failure.

## 3. The Role of Autophagy in Ventricular Remodeling

### 3.1 Definition and Physiological Functions of Autophagy

Autophagy is an intracellular process responsible for the degradation and recycling of damaged or excess organelles, serving as a vital component of the cell’s internal cleaning and recycling system. This process plays a crucial role in maintaining cellular homeostasis, promoting cell survival under nutrient deficiency, and responding to cytotoxic stimuli [82, 83]. This degradation and recycling of unnecessary or dysfunctional cellular components occurs through the formation of autophagosomes, which then fuse with lysosomes to achieve the degradation of its contents.

The physiological functions of autophagy mainly include maintaining cellular survival and homeostasis, cardiac protection, regulating cell death, participating in immune responses, and metabolic regulation [82]. In terms of cardiac protection, autophagy plays a protective role during MI by degrading and recycling cellular components, reducing cell death, and improving cardiac function [84]. Autophagy is also involved in regulating various cell death mechanisms, including apoptosis and necrosis, affecting the balance of cell survival and death. Additionally, it also plays a significant role in immune responses, including the regulation of inflammation and the function of immune cells such as macrophages [85]. In terms of metabolic regulation, autophagy affects processes such as oxidative phosphorylation and glycolysis, which are crucial for energy production and cellular function.

Autophagy also plays an important role in cardiac remodeling [86]. After myocardial infarction, autophagy helps maintain cardiac function by promoting the clearance of damaged cells and reducing scar size [86, 87, 88]. Autophagy interacts with other cell death mechanisms such as apoptosis and necrosis, and its regulation can affect the overall outcome of cardiac remodeling and function [87]. In terms of molecular mechanisms, autophagy involves the formation and removal of autophagosomes [86]. If this process is impaired, it may lead to the accumulation of autophagosomes and cellular dysfunction. Autophagy is also regulated by various signaling pathways, including mechanistic target of rapamycin (mTOR) and adenosine 5’-monophosphate-activated protein kinase (AMPK), which affect cellular metabolism and stress responses [87].

Mitophagy, as a selective form of autophagy, specifically targets damaged mitochondria and is particularly important for maintaining cardiovascular homeostasis due to the high energy demands of the cardiovascular system [88]. As the myocardium is a metabolically active tissue with high oxidative demands, mitochondria play a central role in maintaining optimal cardiac function. The accumulation of dysfunctional mitochondria is involved in the pathophysiology of cardiovascular diseases, including myocardial infarction, cardiomyopathy, and heart failure [88]. After myocardial infarction, the increase in mitophagy and mitochondrial biogenesis indicates that mitochondria play a key role in cardiac remodeling, and enhanced mitophagy repairs mitochondrial homeostasis by clearing abnormal mitochondria [89]. Mitophagy plays a significant role in diseases such as myocardial infarction, heart failure, and atherosclerosis, and its main regulatory pathways have been widely studied, revealing the close connection between mitophagy and cardiovascular diseases [89]. Additionally, autophagy in the tumor microenvironment, under metabolic stress, allows tumor cells to primarily use glycolysis as a source of energy metabolism [90]. Autophagy supplies energy by recycling metabolites to increase their survival rate. The study further emphasizes the importance of autophagy in maintaining cellular homeostasis and responding to different physiological and pathological states [91]. As research on autophagy mechanisms continues to deepen, the regulation of autophagy provides new perspectives and potential therapeutic strategies for the treatment of cardiovascular diseases [92].

### 3.2 The Role of Autophagy in Ventricular Remodeling After Myocardial Infarction

#### 3.2.1 Autophagy and Myocardial Cell Survival and Death

After myocardial infarction, myocardial cells face the severe challenges of ischemia and energy deficiency, and autophagy plays a complex and crucial role in this process. Autophagy is an intracellular degradation and recycling mechanism that maintains intracellular homeostasis by clearing damaged organelles and misfolded proteins [93]. In the context of myocardial infarction, activation of autophagy helps protect myocardial cells by clearing harmful substances that induce myocardial cell death, thus maintaining cell survival and function [94, 95]. However, excessive activation of autophagy can result in uncontrolled degradation of cellular materials, leading to cellular dysfunction and death. This form of cell death, known as Type II programmed cell death, occurs when excessive autophagy exacerbates myocardial injury, further compromising cardiac function [96]. In myocardial infarction models, excessive autophagy activation is associated with decreased myocardial cell survival rates and impaired heart function.

The beneficial effects of autophagy on left ventricular function have also been studied. Research indicates that the activation of autophagy can improve left ventricular function after myocardial infarction. For example, overexpression of miR-99a promotes autophagy, leading to significant improvements in ejection fraction and fractional shortening in mice [97]. Similarly, the upregulation of cathepsin D (CTSD) (a lysosomal protease) enhances autophagic flux, combating cardiac remodeling and dysfunction. In terms of therapeutic intervention, strategies such as adenovirus-mediated hepatocyte growth factor (Ad-HGF) have been found to improve cardiac remodeling and protect cardiac function by promoting autophagy and necroptosis while inhibiting apoptosis [98]. Additionally, delivery of miR-24 after infarction reduces infarct size and improves cardiac function by downregulating pro-apoptotic proteins [99].

The beneficial effects of autophagy on left ventricular function are mediated through various molecular pathways, including the mTOR/P70/S6K (P70/S6K, p70 ribosomal protein S6 kinase) signaling pathway, and involve the regulation of subcellular organelles crucial for myocardial cell function [100]. Clinically, inhibiting myocardial cell apoptosis and promoting autophagy is crucial for reducing cardiac remodeling and improving heart function, highlighting the potential of autophagy-targeted therapeutic strategies in managing heart failure after myocardial infarction [101]. The latest research also indicates that mitophagy, as a selective form of autophagy, plays a key role in clearing damaged mitochondria and maintaining mitochondrial number and function [102]. After myocardial infarction, myocardial cells undergo ischemia and reperfusion injury, accompanied by abnormal mitochondrial function and increased numbers, leading to the formation of myocardial fibrosis. The activation of mitophagy has potential therapeutic value for improving myocardial injury and fibrosis.

#### 3.2.2 Autophagy’s Role in Cardiac Remodeling

**Fig. 3.**
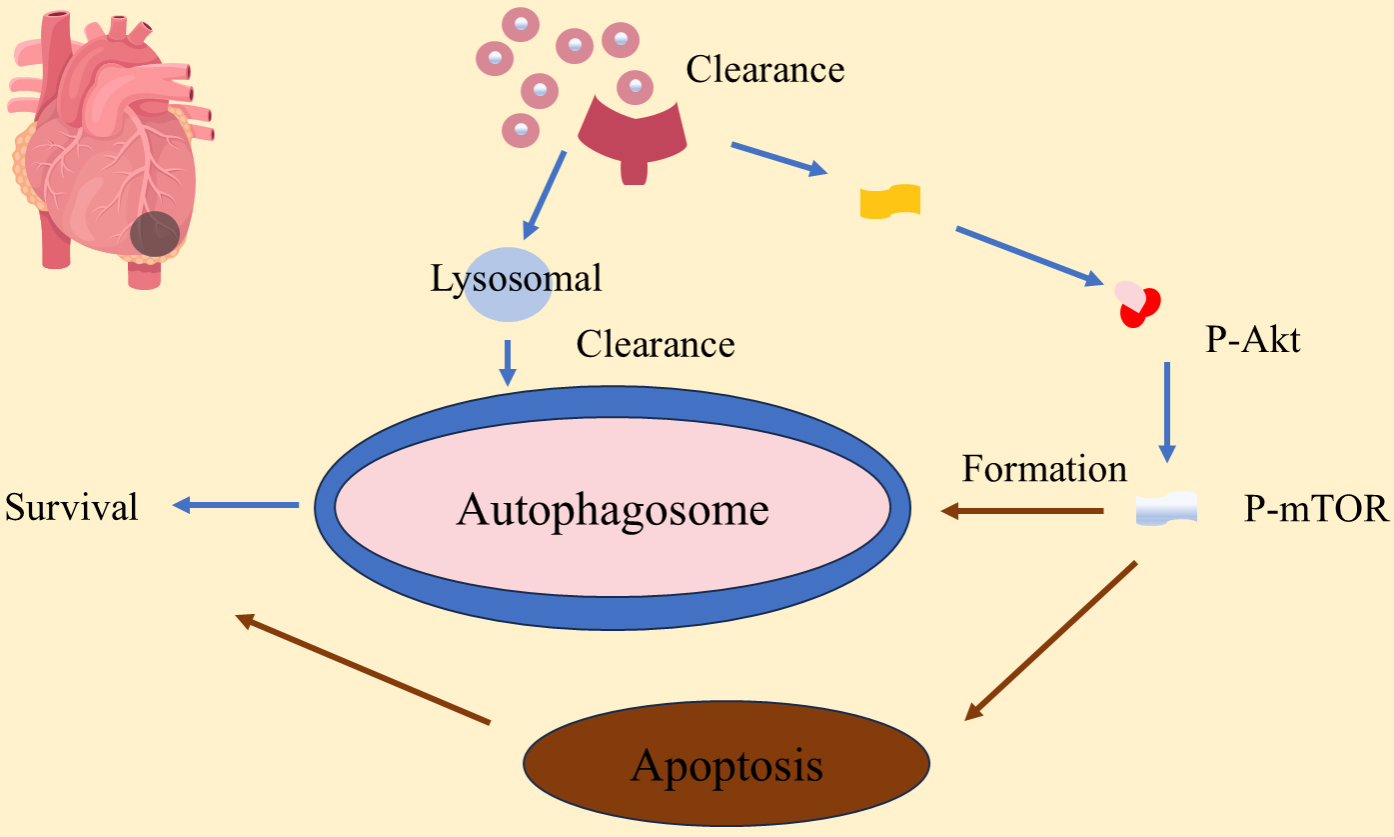
**Autophagy plays a role in myocardial remodeling in myocardial infarction**. P-AKT, phosphorylated protein kinase B; P-mTOR, phosphorylated mammalian target of rapamycin.

Autophagy plays a significant role in the process of cardiac remodeling, protecting myocardial cells by clearing damaged organelles and misfolded proteins [99, 101]. The mechanism of autophagy in myocardial remodeling involves the p-PI3K/Akt (p-PI3K, phosphorylated phosphatidylinositol 3-Kinase; Akt, protein kinase B) signaling pathway and the phosphorylation status of p-mTOR, which together regulate the process of autophagy and affect the survival and apoptosis of cardiomyocytes [99]. When the p-PI3K/Akt pathway is activated in cardiomyocytes, it can promote the formation of autophagosomes by inhibiting mTOR, thereby promoting cell survival. The formation of autophagosomes is a key step in the process of autophagy, which relies on the precise regulation of a series of autophagy-related genes (ATGs) [99]. After myocardial infarction, moderate autophagy clears damaged proteins and organelles, enhances metabolic adaptability, protects cardiomyocytes, and reduces cardiac remodeling. p-mTOR plays a central role in regulating autophagy with its phosphorylation status determining autophagy activation [99, 100]. The mechanism of autophagy in myocardial remodeling is shown in Fig. 3, highlighting its dual effects. While moderate autophagy activation supports myocardial cell survival and cardiac function, excessive autophagy may lead to cellular dysfunction and death [102, 103]. This protective effect of autophagy is especially significant in cardiac function post-myocardial infarction. For instance, overexpression of miR-99a promotes autophagy, improving survival rates and cardiac function in mice after myocardial infarction, possibly through the mTOR/P70/S6K signaling pathway [104]. Additionally, Ad-HGF treatment improves cardiac remodeling, helps maintain cardiac function, reduces scar size, and decreases aggregates in cardiac tissue by promoting autophagy and necroptosis while inhibiting apoptosis [105, 106].

## 4. Discussion and Outlook

After myocardial infarction, the process of autophagy, mitophagy, and mitochondrial function are intricately interconnected and play pivotal roles in ventricular remodeling. It is evident that these processes are not merely passive responses to cardiac injury but actively contribute to the pathogenesis and the development of potential therapeutic interventions. Autophagy and mitophagy, in particular, emerge as double-edged swords, with their moderate activation being cardioprotective and their dysregulation contributing to maladaptive remodeling.

Future research should focus on elucidating the precise molecular mechanisms that govern the balance between the protective and detrimental effects of autophagy and mitophagy. Additionally, there is a pressing need for clinical trials to validate the safety and efficacy of novel therapeutics aimed at modulating these pathways in patients post-myocardial infarction. The advent of precision medicine offers a promising avenue for personalized treatment strategies, where the baseline autophagy and mitophagy profiles of individual patients could inform tailored interventions. Moreover, the intersection of mitochondrial biology with emerging technologies such as gene editing and stem cell therapy opens up new frontiers in regenerative cardiology.

Current therapies for cardiac remodeling have shown moderate results. Identifying new factors, such as autophagy, that regulate inflammation and repair responses may provide new therapeutic targets to prevent adverse remodeling and heart failure [107]. The latest research indicates that exercise can regulate autophagy levels or autophagic flux, improving cardiac function and having a certain therapeutic and guiding effect on cardiovascular diseases [108]. Exercise upregulates mitochondrial autophagy through the FNDC5/Irisin-PINK1/Parkin-LC3II/L-P62 (FNDC5, fibronectin type III domain containing 5; PINK1, PTEN-induced kinase 1; LC3II, microtubule-associated protein 1 light chain 3-II; L-P62, lipidated p62) signaling pathway, enhances antioxidant capacity, inhibits oxidative stress, and improves cardiac function [109]. These findings provide a new perspective for the treatment of cardiac remodeling, emphasizing the importance of autophagy in cardiac remodeling and offering new targets for future therapeutic strategies.

## 5. Conclusion

Left ventricular dysfunction, frequently resulting from post-myocardial infarction remodeling, is a significant risk factor for cardiovascular health, potentially leading to heart failure. Effective management of this condition, incorporating pharmacological, treatments, interventional therapies, and lifestyle changes, is crucial to prevent further remodeling and heart failure. Autophagy and mitochondrial function are crucial in this process. Autophagy when balanced, plays a protective role, but excessive activation can lead to detrimental effects. Given the central role of mitochondria in cardiac remodeling, regulating these processes offers promising strategies for treatment. The content summary of this article is presented in Table 1 (Ref. [11, 12, 13, 14, 19, 20, 21, 22, 25, 26, 27, 28, 31, 32, 33, 34, 35, 36, 37, 38]).

**Table 1.** **Overview of autophagy, mitophagy, mitochondrial roles, and therapeutic strategies in ventricular remodeling post-myocardial infarction**.

Category	Subcategory	Description	Impact on Ventricular Remodeling	References
Autophagy	Without MI	Maintains cellular energy and clears damaged organelles	Contributes to baseline cardiac function and homeostasis	[11, 12]
	With MI	Enhanced to clear dysfunctional mitochondria and damaged proteins	Protects against ischemic injury by reducing cell death, influences remodeling	[13, 14]
Mitophagy	Without MI	Clears damaged mitochondria to maintain energy homeostasis	Ensures optimal cardiac performance by maintaining mitochondrial quality	[19, 20]
	With MI	Increased to repair mitochondrial dysfunction post-MI	Essential for post-MI recovery by clearing abnormal mitochondria	[21, 22]
Mitochondria	Without MI	Central to myocardial function	Manages energy production and oxidative stress under normal conditions	[25, 26]
	With MI	Impaired post-MI, leading to cell death and dysfunction	Increased oxidative stress and apoptosis contribute to cardiac remodeling	[27, 28]
Therapeutic Strategies	Inhibition of mitochondrial oxidative stress	Reduces oxidative damage to mitochondria	Prevents heart failure and improves cardiac function	[31, 32]
	Promotion of fundc1-mediated mitophagy	Enhances clearance of damaged mitochondria	Restores mitochondrial function and protects from ischemic injury	[33, 34]
	Pharmacological inhibition of autophagy	Controls autophagy levels to prevent excessive degradation	Reduces cardiac remodeling and improves post-MI outcomes	[35, 36]
	Exercise rehabilitation	Modulates autophagy and mitophagy	Improves cardiac function and limits adverse remodeling	[37, 38]

MI, Myocardial infarction.
